# Rising premature menopause and variations by education level in India

**DOI:** 10.1038/s41598-024-67730-2

**Published:** 2024-08-06

**Authors:** Karan Babbar, Vanita Singh, M. Sivakami

**Affiliations:** 1 Centre for Development Studies, Jindal Global Business School, OP Jindal Global University, Sonipat, India; 2 Economics and Public Policy, Management Development Institute, Gurugram, India; 3 Center for Health and Social Sciences, School of Health Systems Studies (SHSS), Tata Institute of Social Sciences (TISS), Mumbai, India

**Keywords:** Menopause, Premature menopause, Mid-life experience, Gender, Education, Decomposition, NFHS-5, India, Health care economics, Health policy, Public health

## Abstract

The proportion of women experiencing premature menopause is on the rise in India, particularly in the age groups of 30–39 years. Consequently, there is a need to understand the factors influencing the prevalence of premature menopausal status among women. Our study uses the data from 180,743 women gathered during the latest Indian version of the Demography Health Survey (National Family Health Survey-5). Our results suggest that close to 5% of women in rural areas and 3% of women in urban areas experience premature menopause, and this figure varies across Indian States. The regression results show that surgical menopause, lower levels of education, poorer wealth index, rural residence, female sterilization, and insurance coverage are key drivers of premature menopause. One of the striking factors is that the prevalence of premature menopause among those with the lowest levels of education (6.85%) is around seven times higher than those with the highest level of education (0.94%). We conducted a decomposition analysis to delve into the factors contributing to this inequality. The results show that undergoing a hysterectomy (surgical menopause) account for 73% of the gap in premature menopausal rates between women with the lowest and highest levels of education. This indicates that women with poor education are more likely to undergo hysterectomy at a younger age. This finding warrants further exploration as we would expect that women from lower socio-economic background would have limited access to surgical care, however, our results suggest otherwise. This perhaps indicates a lack of awareness, lack of alternative treatment options, and over-reliance on surgical care while neglecting conservative management. Our results have implications for addressing the diverse needs of the increasing number of women in their post-menopause phase and for focusing on conservative treatment options for these women.

## Introduction

For women worldwide, menopause represents a critical biological transition marked by range of physical and psychological changes. While it is a natural process, this phase can be challenging due to associated changes, potentially leading to distress^1–4^. Women may be more vulnerable during the post-menopause phase as a result of menopause and its related morbidities^5^. Until recently, issues related to menopause were a neglected research area, especially in developing countries. However issues related to menopause are gaining attention as women spend a significant proportion of their life in post-menopause phase due to increased life expectancy^6,7^. Specifically, premature menopause is of significant concern given its association with increased morbidity and mortality risks for women.

Premature menopause, that is menopause before the age of 40 years^8^ is of primary concern given its adverse effects on cognition, cardiovascular, bone, mood, and sexual health^9,10^. Premature menopause is either due to surgical procedures (hysterectomy or oophorectomy) or primary ovarian insufficiency^8,10^. Premature menopause is associated with increased morbidity and mortality risks for females and, thus, requires the attention of health policy makers. With improvements in healthcare systems and overall economic development, women in South-East Asia (SEA) enjoy higher life expectancy, but, at the same time, issues of premature menopause render these women vulnerable to increased morbidity risks during the postmenopausal phase. Thus, issues of premature menopause in SEA region are emerging as a significant public health concern.

The onset of menopause varies across geographies. Numerous research findings suggest that women residing in developing nations undergo natural menopause several years earlier than their counterparts in developed countries^11,12^. Further, a recent trend analysis study reports an increasing prevalence of premature menopause, especially in SEA^13^. There is an overall decline in mean age at menopause except in North Africa/ West Asia and Europe^13^. Premature menopause is an emerging issue in South-East Asia, including India, and warrants the attention of researchers and policy makers alike. Whether contextual and socio-economic factors could explain the geographical variations in the age at menopause is not clear and requires further exploration.

Premature menopause, which is linked to morbidity and mortality risk, has been the topic of study by many researchers across the globe^5,14,15^. These studies explore various factors contributing to premature menopause, including lifestyle, demographic, cultural and environmental^16–19^. Among all the factors, a few have been consistently found to be associated with menopause, though the direction of association remains inconsistent. Some studies have identified lower socio-economic status, lower educational status and rural residence as predictors of premature menopause^6,20–22^. Interestingly, conflicting findings emerge, with other studies linking rural residence to a later onset of menopause^23^. One of the primary reasons for premature menopause is surgical menopause^24,25^.

Existing studies have focused on the predictors of age at menopause and focus on a single state, while nationally representative studies are limited. Few nationally representative studies^6^ that do exist draw on earlier NFHS data sets, spanning almost two decades back. Our study addresses this research gap by using the latest round of NFHS and focusing on the variations in the proportion of menopausal women across Indian States. In India, formal education about menopause is non-existent. Studies conducted in different parts of the world underline that women in the menopausal stage often possess limited knowledge about menopause, and even if aware, they lack an understanding of its consequences^26–28^. Thus, we use decomposition analysis to understand the factors contributing to the differences in the proportion of premature menopause based on education levels.

## Methodology

### Data source

We use the data from the latest Indian version of the Demographic Health Survey, also popularly known as the National Family Health Survey-5 (NFHS-5) conducted between 2019 and 2021, that are freely available in the public domain. The NFHS-5 survey is conducted by the Ministry of Health and Family Welfare in collaboration with the International Institute of Population Sciences, Mumbai. This survey utilizes four questionnaires: the biomarker, household, men and women’s questionnaire. Our study focuses on the women's questionnaire, which gathers information on the characteristics and reproductive health of women in the households, and the household questionnaire, which collects data on the characteristics and reproductive health of all household members. The NFHS-5 data consists of information gathered from 724,115 women aged 15–49 and 101,839 men during the 636,699 household interviews conducted across the former 29 states and seven union territories. Our study focused on women aged 30 to 39 who were currently not pregnant or non-amenorrhoeic or those who have not menstruated since their last birth^6^. These conditions restricted our NFHS-5 data to 180,743 females aged 30 to 39. The NFHS-5 survey collects data for women aged 15–49; however, for the specific study on premature menopause, a narrower age range is necessary to ensure reliable and relevant findings. Therefore, we restricted the age range to women between 30 and 39 years old. Menopause typically occurs in women around the age of 45–55, with the average age of onset being 51. Focusing on women aged 30–39 allows us to capture a population that is not likely to be experiencing or approaching menopause. Additionally, including women below the age of 30 may introduce unrelated factors and impede our ability to draw meaningful conclusions about menopausal transitions.

### Dependent variable

The following questions have been considered to identify the dependent variables for this study using the NFHS-5 questionnaire:

The question asked to women was, “When did your last menstrual period start?” Women who reported having menstruated within the past 12 months were categorized as not being in menopause. On the other hand, women who had not menstruated for more than 12 months, were currently in menopause, or had undergone hysterectomy were classified as menopausal. We converted this into a binary variable called “*menopause*”. It is coded as ‘1’ if women had not menstruated for more than 12 months, were currently in menopause, or had undergone hysterectomy, and coded as ‘0’ for women who reported having menstruated within the past 12 months. This question was limited to women aged 30 to 39 years to focus on premature menopause.

### Independent variables

Based on the literature review, we have used several independent variables in the study, which have been segregated as socio-demographic, nutritional and biological factors.

- Socio-Demographic Factors include Education Level of Respondents (No Education, Primary, Secondary & Higher), Wealth Index (Poor, Middle & Rich), Caste (General, Other Backward Classes, Scheduled Caste, Scheduled Tribe), Religion (Hindu, Muslim & others), & Area of Residence (Urban & Rural).
- Nutritional and Biological Factors include the age of women (30–34 years, 35–39 years), children ever born (0, 1–2, 3–4, 5 and above), age of women at first birth (less than 18 years, 18–21 years, 22–24 years, 25 & above), age of women at last birth (less than 21 years, 22–24 years, 25–28 years, 29 & above), contraceptive usage (no, yes), experienced foetal loss (no, yes), body mass index (Underweight: less than or equal to 18.5 kg/m^2^, Normal: 18.5–24.9 kg/m^2^, Overweight: more than or equal to 25 kg/m^2^), anaemia status (non-anaemic, anaemic).

## Data analysis

Data analysis was performed using Stata Version 15.1. We employed a binary logistic regression model to examine the socio-demographic, nutritional and biological factors that influence the premature menopausal status of women aged 30–39 years.

The estimated model is shown below:

$$logit \left(p\right)= log(\frac{p}{1-p})={\beta }_{0}+{\beta }_{1}{X}_{1}+{\beta }_{2}{X}_{2}+\dots +{\beta }_{n}{X}_{n}$$

where *p* represents the probability of the binary response variable Y, which has two categories (0 = non-menopausal women, and 1 = menopausal women), *β*_*1,*_* β*_*2,*_* β*_*n*_ are the coefficients of independent variables X_1_, X_2_ up to X_n_. In other words, we compare women who are currently in menopause with those who are not. In this specification, the dependent variable is binary and hence, binary logit regression was used to produce the estimates. One potential issue with this model could be that individuals within the same district may be more similar to each other than to individuals in different districts in terms of geographical proximity, shared socio-cultural influences, or local policies and practices. Thus, district fixed effects were included in the models. All the standard errors were clustered at the district level.

In the second stage of our enquiry, we focused on understanding the differences in premature menopause levels between women with different levels of education. To do so, we utilized the modified form of Blinder-Oaxaca (B-O) decomposition, which is appropriate for binary outcomes^29^. Following Babbar & colleagues, we used the B-O decomposition to analyse the disparities in premature menopause by the education levels^30^. Originally, the B-O decomposition utilized linear regression modelling for continuous variables, offering an approach to elucidate disparities between two groups in final outcomes. These groups may vary based on factors like age, wealth index, caste, and religion. Our study aims to comprehend disparities in premature menopause among women with different levels of education.

To do so, two separate groups were created i.e., women with no education vs women with higher education. Differences between these groups, assessed through BO methods, are bifurcated into two components. The initial difference segment arises from variations in mean values of the independent variable between groups. The remaining disparity, unaccounted for by such differences, constitutes the second part. However, this method is inadequate for binary outcome variables, as in our case. Hence, we employed Stata’s “*fairlie*” package for decomposition analysis, specifically to elucidate the disparities in premature menopause between the women with no education vs those with the highest education. All data analyses were conducted using Stata 15.1.

## Results

### Onset of menopause in India

Table 1 presents data on the proportion of women in menopause by age categories, among women aged 30 to 49, both nationally and in major states. The data in Table 1 reveals that 14.66% of women in this age group are in menopause nationwide. Additionally, the percentage of women in menopause is lower in urban areas (12.19%) compared to rural areas (15.96%). The percentage of women in premature menopause (menopause before 40 years) is higher in rural areas (about 4.73%) in comparison to urban areas (about 2.55%).

**Table 1:** Percentage of currently married women aged 30–49 in Menopause by age, NFHS-5 (2019–21).

India/State	Age							
	30–34	35–39	40–41	42–43	44–45	46–47	48–49	Overall
India-Urban	1.23	3.74	8.99	12.98	21.71	30.14	46.14	12.19
India-Rural	2.96	6.43	13.3	18.14	28.13	37.1	50.62	15.96
India-Total	2.36	5.5	11.81	16.33	25.96	34.64	49.1	14.66
State								
Andhra Pradesh	5.47	11.11	24.34	27.21	38.89	49.69	63.31	23.94
Telangana	5.02	12.11	21.02	29.34	40.92	48.41	63.62	23.84
Bihar	8.41	13.01	20.57	25.97	37.4	46.24	56.89	21.94
Assam	1.52	3.63	12.14	19.76	32.08	45.86	56.62	16.18
Odisha	0.79	3.78	12.36	19.02	29.09	42.93	57.37	15.48
Uttar Pradesh	2.18	5.7	12.64	19.05	28.54	38.67	51.36	15.31
West Bengal	0.97	3.38	9.92	13.76	24.66	37.83	53.04	15.27
Jharkhand	2.73	5.88	12.2	19.28	25.98	37.88	50.34	14.96
Tripura	0.65	2.08	8.44	11.1	29.86	47.65	62.21	14.68
Ladakh	1.31	7.39	8.31	13.93	25.14	47.65	49.93	14.44
Gujarat	1.91	6.33	12.84	18.47	25.31	29.99	46.01	14.15
Chhattisgarh	1.14	3.94	10.32	15.78	29.23	35.85	52.52	13.68
Madhya Pradesh	2.4	5.28	10.57	14.91	23.46	31.03	44.66	13.09
Goa	0	2.54	4.22	5.9	20.11	28.78	45.27	12.62
Arunachal Pradesh	1.49	3.37	11.53	11.82	21.84	32.25	43.87	12.52
Punjab	1.19	4.48	9.95	12.71	21.23	31.17	46.7	12.34
Karnataka	2.69	5.12	9.09	12.38	22.08	25.22	38.35	12.05
Tamil Nadu	0.91	2.26	6.52	12.8	20.55	26.68	46.78	11.99
Maharashtra	1.3	4.3	9.91	12.15	19.76	32.51	43.11	11.98
Nagaland	2	3.2	6.66	10.35	15.09	24.19	44.36	11.96
Uttarakhand	1.14	3.39	10.1	14.73	25.03	30.3	42.87	11.95
Delhi	0.54	3.15	10.33	12.2	22.52	35.05	51.45	11.72
Haryana	1.23	3.86	8.09	11.16	22.53	29.96	43.19	11.04
Rajasthan	1.62	3.99	7.3	12.14	18.8	24.5	40.95	10.92
Meghalaya	1.61	2.48	6.24	8.3	20.39	33.85	41.15	10.21
Manipur	1.04	2.87	6.84	7.89	15.36	30.4	45.08	10.02

All numbers are in percentages. The table above represents only those states where the percentage of women currently in menopause are 10 & above.

Andhra Pradesh, Telangana, and Bihar emerge as the top three states in India for menopause levels among women aged 30–49. However, when we examine menopausal rates in the age groups 30–34 and 35–39, Bihar’s numbers exceed those of Andhra Pradesh and Telangana. In fact, the prevalence rates of premature menopause in Bihar are almost four times and two times the national average in the age groups 30–34 and 35–39, respectively.

Based on the descriptive statistics data shown above, our study aims to understand two main objectives. First, given that menopause is primarily influenced by biological factors, the significant variations observed among states are somewhat unexpected and warrant further investigation. Therefore, it is necessary to explore the potential impact of non-biological factors, such as socio-economic variables, on menopause. Second, based on the numbers for premature menopause, it is important to understand the various factors influencing the women who experience premature menopause as compared to those not experiencing menopause. Thus, in the second part of the study, we explore the socio-demographic, biological and reproductive factors affecting the premature menopause of women.

### Sample characteristics

Based on the previous research, it is evident that both socio-demographic factors and nutritional and biological factors play a significant role in the onset of premature menopause. Therefore, to provide a comprehensive understanding, we now describe the sample characteristics pertaining to socio-demographic factors as well as nutritional and biological factors.

Descriptive statistics for the socio-demographic, nutritional & biological factors are presented in Table 2. The study uses data from 180,743 women aged 30 to 39 who participated in the NFHS-5 survey. The sample consists of approximately 34 percent of women living in urban areas and the remaining in rural areas. The majority of the sample, around 84 percent, follows the Hindu religion, followed by Muslims (10%) and other religious groups (6%). In terms of caste, approximately 45 percent of the women belong to the OBC category, followed by General (22%), SC (23%), and ST (10%). In terms of wealth index, around 57 percent of the women belong to the poor and middle wealth index category, while the remaining 43 percent belong to the rich wealth index category.

**Table 2:** Respondent’s characteristics of women aged 30–39 years, NFHS-5 (2019–21).

Respondent characteristics	Overall sample
Variables	Frequency (percentage)
Socio-demographic factors	
Education	
No Education	51,930 (27.98)
Primary	27,241 (15.00)
Secondary	80,298 (43.62)
Higher	21,274 (13.39)
Wealth Index	
Poor	75,149 (36.90)
Middle	37,572 (20.32)
Rich	68,022 (42.78)
Caste	
General	36,495 (22.23)
Scheduled Caste	36,123 (22.66)
Scheduled Tribe	35,144 (9.69)
Other Backward Classes	72,981 (45.42)
Religion	
Hindu	140,592 (84.01)
Muslim	17,306 (10.49)
Others	22,845 (5.50)
Area of Living	
Urban	47,357 (34.33)
Rural	133,386 (65.67)
Nutritional & biological factors	
Age Group	
30–34	89,840 (49.71)
35–39	90,903 (50.29)
Children ever born	
0	11,291 (5.46)
1–2	92,823 (53.81)
3–4	63,498 (33.76)
5 +	13,131 (6.97)
Age of women at first birth	
Less than 18	31,055 (18.51)
18–21	55,143 (31.01)
22–24	43,544 (24.01)
24 and above	51,001 (26.46)
Age of women at last birth	
Less than 21	23,095 (14.42)
22–24	38,531 (22.24)
25–28	56,411 (31.17)
29 & above	62,706 (32.17)
Contraceptive usage	
No	47,727 (24.84)
Yes	133,016 (75.16)
Body mass index	
Underweight	19,994 (10.91)
Normal	103,362 (54.18)
Overweight	57,387 (34.91)
Anaemia	
Non-anaemic	85,168 (46.89)
Anaemic	95,575 (53.11)
Health insurance	
No	117,766 (67.36)
Yes	62,977 (32.64)
Female sterilization	
No	101,655 (53.40)
Yes	74,473 (46.60)

Numbers mentioned in the table are the actual frequencies of the various socio-demographics, whereas percentage are calculated using the appropriate weights.

The sample is evenly distributed across all age groups. Nearly half of the sample has 1–2 children. Around 26 percent and 32 percent of the women in our sample were 24 years old or above and 29 years old or above during their first and last birth, respectively. Approximately 75 percent of the sample uses contraception, and 54 percent have a normal body mass index (BMI). Additionally, 53 percent of our sample is classified as anaemic.

Table 3 reports the results on the number of women aged 30–39 currently in premature menopause reported by the sample characteristics in the study. The results show interesting patterns related to socio-demographic factors, including education level of respondents, wealth index, caste, religion, and area of living. It shows that the percentage of women who attained menopause were highest in those with no education (7%) and lowest in those with higher education (1%). The percentage of women in menopause decreases as the wealth increases, with 5% of women from poor and middle backgrounds experiencing menopause compared to only 3% from the rich quantile. The percentage of women in premature menopause remains similar across different castes and religions. We also find that a significantly higher proportion of women surveyed in rural areas are in premature menopause than women in urban areas.

**Table 3:** Percentage of women in menopause by background characteristics of women aged 30–39, NFHS-5 (2019–21).

Respondent characteristics	Respondents currently in menopause	*p*-value
Variables	(%)	
Socio-demographic factors		
Education		
No Education	3192 (6.85)	< 0.01
Primary	1067 (4.45)	
Secondary	2222 (2.92)	
Higher	193 (0.94)	
Wealth index		
Poor	3177 (5.11)	< 0.01
Middle	1605 (4.52)	
Rich	1892 (2.76)	
Caste		
General	1108 (3.25)	< 0.01
Scheduled caste	1420 (4.05)	
Scheduled tribe	936 (3.36)	
Other backward classes	3210 (4.44)	
Religion		
Hindu	5517 (4.09)	< 0.01
Muslim	624 (3.66)	
Others	533 (2.98)	
Area of living		
Urban	1178 (2.55)	< 0.01
Rural	5496 (4.73)	
Nutritional & biological factors		
Age group		
30–34	1969 (2.40)	< 0.01
35–39	4705 (5.55)	
Children ever born		
0	273 (2.82)	< 0.01
1–2	2920 (3.13)	
3–4	2838 (5.11)	
5 +	643 (6.01)	
Age of women at first birth		
Less than 18	2306 (7.73)	< 0.01
18–21	2402 (4.65)	
22–24	1179 (2.82)	
24 and above	787 (1.64)	
Age of women at last birth		
Less than 21	1728 (7.44)	< 0.01
22–24	1862 (4.96)	
25–28	1856 (3.47)	
29 & above	1228 (2.25)	
Contraceptive usage		
No	3355 (7.71)	< 0.01
Yes	3319 (2.75)	
Body mass index		
Underweight	827 (4.59)	< 0.01
Normal	3620 (3.80)	
Overweight	2227 (4.08)	
Anaemia		
Non-anaemic	3561 (4.53)	< 0.01
Anaemic	3113 (3.50)	
Health insurance		
No	4124 (3.79)	< 0.01
Yes	2550 (4.38)	
Female sterilization		
No	3524 (3.82)	< 0.01
Yes	3094 (4.27)	

Examining biological and nutritional factors, we find that age group, number of children ever born, age of women at first & last birth, usage of contraceptives, BMI and anaemic levels predicts the premature menopause. The percentage of women in the premature menopause increases with the increase in age, with only 2.4% of the women aged 30–34 experiencing premature menopause compared to 5.5% of women aged 35–39. Similarly, the percentage of women in menopause increases with the increase in the number of children ever born, as 3% of women with zero or 1–2 children are in menopause compared to 6% of women with 5 or more children. Additionally, women who had their first and last births at younger ages (less than 18 and 21 years, respectively) have a higher percentage of menopause (7.73% and 7.44%) compared to those who had their first and last births at older ages (greater than 24 and 29 years, respectively) with lower percentages (1.64% and 2.25%). Interestingly, women who do not use contraceptives, are overweight or underweight and are non-anaemic show a higher proportion of experiencing menopause.

### Odds ratio for the determinants of premature menopause

Col (1–2) of Table 4 presents the odds ratio from the binary logistic regression model, examining the likelihood of women experiencing premature menopause compared to those who are not without and with district fixed effects, respectively. Odds ratios greater than 1 indicate an increased likelihood of experiencing premature menopause, while odds ratios less than 1 suggest a decreased likelihood. Below, we highlight our most preferred results from column 2. Interestingly, women with a history of surgical menopause emerge as the primary contributors to premature menopause, exhibiting an exceptionally high odds ratio (OR = 1020.16, *p* < 0.01). Women with higher levels of education were less likely to experience premature menopause compared to those with no education (OR = 0.429, *p* < 0.01), suggesting an inverse relationship between education and premature menopause. Women in the richest wealth quantile had lower odds of experiencing premature menopause compared to those in the poor wealth quantile (OR = 0.847, *p* < 0.01). There was no significant impact of religion, caste, and area of living on women’s premature menopausal status.

**Table 4:** Results from binary logit regression for the onset of premature menopause among women aged 30–39, NFHS-5 (2019–21).

Variables	Odds ratio	Odds ratio
Surgical menopause	671.05***	1020.166***
Socio-demographic factors		
Education level of respondents		
No education		
Primary	0.697***	0.733***
Secondary	0.652***	0.672***
Higher	0.412***	0.429***
Wealth index		
Poorest		
Middle	0.952	1.000
Richest	0.788**	0.847**
Caste		
Scheduled Caste		
Scheduled Tribe	0.957	0.973
Other Backward Class	1.016	0.977
General	1.041	1.018
Religion		
Hindu		
Muslim	0.917	0.920
Others	0.905	0.969
Area of living		
Urban		
Rural	0.918	0.932
Reproduction & Nutritional Factors		
Age groups		
30–34		
35–39	2.487***	2.587***
Number of children		
0		
1–2	0.309***	0.319***
3–4	0.311***	0.320***
5 +	0.370***	0.375***
Age of women at first birth		
Less than 18		
18–21 years	0.694***	0.688***
22–24 years	0.592***	0.597***
25 and above	0.498***	0.500***
Age of women at last birth		
Less than 21		
21–24 years	0.854*	0.820**
25–28 years	0.774**	0.724***
29 years and above	0.511***	0.480***
Contraceptive usage		
No		
Yes	0.208***	0.175***
Body mass index		
Underweight		
Normal	0.723***	0.730***
Overweight	0.791**	0.792**
Anaemia		
Non-Anaemic		
Anaemic	0.971	0.952
Health insurance		
No		
Yes		
	1.149**	1.129**
Female sterilization		
No		
Yes	1.870***	2.156***
District fixed effects	No	Yes
Pseudo *R*^2^	0.6457	0.6652
Observations	176,128	171,869

Examining the nutritional and biological factors, we found that women in the age group 35–39 had higher odds of experiencing menopause compared to those aged 30–34 (35–39 years: OR = 2.587, *p* < 0.01). Women with a higher number of children had lower odds of experiencing premature menopause compared to those with no children (1–2 children: OR = 0.319, *p* < 0.01; 3–4 children: OR = 0.320, *p* < 0.01; 5 + children: 0.375, *p* < 0.01). Consistent with these findings, we also found that the women with higher age at first (25 years and above: OR = 0.500, *p* < 0.01) and last birth (OR = 0.480, *p* < 0.01) had lower odds of experiencing premature menopause compared to those with lower age. Additionally, women who used contraceptives had lower odds of experiencing premature menopause (OR = 0.175, *p* < 0.01), as did women who were normal (OR = 0.730, *p* < 0.01) or overweight (OR = 0.792, *p* < 0.05). Women with health insurance (OR = 1.129, *p* < 0.05), and female sterilization had had higher chances of being in premature menopause (OR = 2.156, *p* < 0.01) compared to those with no health insurance or those who are not sterilized.

### Results from Fairlie decomposition analysis

The summary results from the Fairlie Decomposition Analysis are presented in Table 5, which provides us insights into the predictors contributing to the gap in the premature menopause among women with different levels of education. The mean prediction for the women in the premature menopause was 6.19% for those with highest education, compared with 0.96% for those with the lowest education. This stark of almost six and half fold division raises questions and warrants further investigation. The decomposition analysis reveals that around 88% of this gap for women in premature menopause can be explained by the factors included in our analysis. However, around 12% of the gap remains unexplained by the independent variables included in the study, suggesting the presence of other factors not captured by the NFHS-5 dataset, used for this study.

**Table 5:** Summary results of Fairlie decomposition showing the mean differences in premature menopause between women across education levels in India, NFHS-5 (2019–2021).

Mean number of women across Education Levels in Premature menopause	% differences
No education (1)	6.19
Primary education (2)	3.94
Secondary education (3)	2.79
Higher education (4)	0.96
Any education (5)	2.75
Raw differential between women with higher and no education [4–1]	5.23
% Explained	87.99
% Unexplained	12.01
Raw differential between women with primary and no education [2–1]	2.25
% Explained	76.21
% Unexplained	23.78
Raw differential between women with secondary and no education [3–1]	3.39
% Explained	83.59
% Unexplained	16.41
Raw differential between women with any education and no education [5–1]	3.44
% Explained	83.60
% Unexplained	16.40

Next, we look at the detailed decomposition analysis, where we examined the percentage contribution of the numerous factors contributing to the disparities in women’s menopausal stage. Of the explained gap of 88%, around 84% can be attributed to the surgical menopause, followed by age at first birth (11%), and wealth index (5%). This substantial portion underscores the significance of insufficient information due to poor education, potentially leading to surgical menopause and subsequently influencing women's early menopausal status.

### Robustness checks

To ensure the validity of our results, we conducted additional robustness checks. First, we assessed the validity of our results across different educational levels. Specifically, we examined the primary factors influencing premature menopause for women with: (a) secondary vs. no education; (b) primary vs. no education; (c) any education vs. no education. The categories of primary and secondary education were derived from the NFHS-5 survey. Additionally, we introduced a new binary variable, termed “*any education*,” which takes the value of 1 if a woman has attained either primary, secondary, or higher education, and 0 otherwise. The decomposition analysis reveals that around 76–83% of the gap for the women in premature menopause can be explained by the factors included in our analysis (See Table 5). Our findings remained robust, with surgical menopause consistently emerging as the predominant predictor, contributing between 87 and 92% to premature menopause across varying levels of education for women (Tables 6 and 7).

**Table 6:** Fairlie decomposition of average gap on premature menopause between highest and lowest education of women in India, NFHS-5 (2019–21).

Variables	Coefficient	*p*-value	LLCI	ULCI	Percentage contribution (%)
Hysterectomy	0.039	0	0.03793	0.03945	84
Age at first birth	0.005	0	0.00319	0.00719	11
Wealth index	0.002	0.031	0.0002	0.00404	5
Age	0.002	0	0.00111	0.00244	4
Female sterilization	0.002	0.004	0.00053	0.00291	4
Age at last birth	0.001	0.003	0.00049	0.00229	3
BMI	0.000	0.1	− 9E−05	0.00099	1
Religion	0.000	0.167	− 4E−05	0.00022	0
Health insurance	0.000	0.426	− 8E−05	0.00019	0
Anaemia	0.000	0.769	− 0.0002	0.00015	0
Caste	0.000	0.247	− 0.001	0.00025	− 1
Area of living	0.000	0.591	− 0.0018	0.00104	− 1
Number of children	− 0.002	0.004	− 0.0033	− 0.0006	− 4
Contraceptive usage	− 0.003	0	− 0.004	− 0.0014	− 6

**Table 7:** Robustness: Fairlie Decomposition of average gap on premature menopause between different levels of education of women in India, NFHS-5 (2019–21).

Variables	Percentage contribution		
	R1: Secondary vs no education (%)	R2: Primary vs no education	R3: Any vs no education
Hysterectomy	86.88	91.81	87.07
Age at first birth	6.31	1.94	6.80
Wealth index	4.56	2.67	4.08
Age	3.24	3.07	3.94
Female sterilization	1.67	− 0.16	1.87
BMI	1.16	0.93	1.06
Contraceptive usage	0.91	4.89	− 0.82
Religion	0.75	0.88	0.72
Health insurance	0.05	− 0.11	0.05
Anaemia	− 0.03	− 0.01	− 0.04
Age at last birth	− 0.03	− 1.99	0.52
Area of living	− 0.59	− 0.30	− 0.60
Caste	− 0.78	− 0.40	− 0.72
Number of children	− 4.09	− 3.23	− 3.95

The percentage highlighted in the above table represents the contribution of each variable towards the disparities in the early menopausal levels among women with the different levels of education. Any education is a new binary variable, which takes the value 1 if the women has received primary or secondary or higher education and 0 otherwise.

In the next set of robustness check, we added additional variables which could affect the premature menopausal status of women, as shown in Table 8. There is a growing literature that mass-media affects sexual and reproductive health outcomes, including menopausal status of women^31^ . In the first column, we added mass-media characteristics. The frequency of newspaper reading, radio listening, and television watching resulted in the formation of a novel variable termed “mass media exposure.” This variable assumes a value of 0 when a respondent has no exposure to any of the media. Likewise, it assumes values of 1 and 2 if the respondent's exposure to these media items is less than once a week and at least once a week, respectively.

**Table 8:** Robustness check: logit regression results on premature menopause after inclusion of additional control variables, NFHS-5 (2019–21).

	(1)	(2)	(3)
Variables	Media	Women's Chars	All
Surgical menopause	1,023***	10,552***	11,028***
Mass-media			
No			
Partial	0.836**		0.564
Full	0.923		0.740
Working status of women			
No			
Yes		0.574**	0.578**
Mobile phone usage			
No			
Yes		1.004	0.962
Bank account			
No			
Yes		0.598	0.599
Internet usage			
No			
Yes		0.863	0.870
Socio-Demographic Factors			
Education level of respondents			
No Education			
Primary	0.747***	0.815	0.830
Secondary	0.688***	1.879*	2.010**
Higher	0.438***	0.415	0.442
Wealth index			
Poor			
Middle	1.015	1.796*	1.893*
Rich	0.860*	1.897*	1.999*
Social category			
Scheduled Caste			
Scheduled Tribe	0.971	0.672	0.674
Other Backward Classes	0.977	0.911	0.910
General	1.018	0.688	0.685
Religion			
Hindu			
Muslim	0.917	0.689	0.687
Others	0.968	1.865	1.853
Area of living			
Urban			
Rural	0.931	2.061**	2.090**
Reproduction & Nutritional Factors			
Age of respondents			
30–34			
35–39	2.590***	3.001***	3.005***
Number of children			
0			
1–2	0.319***	0.429	0.409
3–4	0.319***	0.521	0.488
5 +	0.373***	0.519	0.470
Age at first birth			
Less than 18			
18–21 years	0.687***	0.758	0.771
22–24 years	0.596***	0.452*	0.464*
25 and above	0.500***	0.519	0.511
Age at last birth			
Less than 21			
21–24 years	0.821**	0.589	0.573
25–28 years	0.724***	0.801	0.784
29 and above	0.479***	0.485	0.479
Contraceptive usage			
No			
Yes	0.176***	0.0927***	0.0925***
Body mass index			
Underweight			
Normal	0.732***	0.415**	0.422**
Overweight	0.794***	0.442*	0.453*
Anaemia			
Non-Anaemic			
Anaemic	0.953	1.067	1.075
Female sterilization			
No			
Yes	2.158***	3.361***	3.377***
Health insurance			
No			
Yes	1.131**	1.527	1.515
Pseudo R^2^	0.6653	0.7629	0.7636
Observations	171,869	7,682	7,682

Another important factor influencing the premature menopause of women is labor force participation, which provides them a degree of control over their life including crucial health decisions. Additionally, the usage of mobile phones, having bank account and internet usage could also contribute to access to health information and services, fostering a proactive approach towards reproductive health. In column 2, we added additional variables including, working status, mobile phone usage, bank account, internet usage. In the last column, all these variables were added to understand the determinants of premature menopause for women in our sample.

Across all three columns, our main results remain consistent, and surgical menopause consistently emerges as the most important predictor, with odds ratio ranging from 1023 when controlling for mass-media to 11,028 when controlling for all additional variables. Subsequently, a detailed decomposition analysis was done after controlling for all these variables, as shown in Table 9. Our findings remained robust, with surgical menopause consistently emerging as the predominant predictor, contributing between 85 and 95% to premature menopause across varying levels of education for women. These results serve to further substantiate our findings and enhance their overall robustness.

**Table 9:** Robustness check: Fairlie decomposition of average gap on premature menopause between highest and lowest levels of education of women after inclusion of additional covariates, NFHS-5 (2019–21).

Variables	Percentage contribution		
	Mass-media	Additional vars	All
Hysterectomy	84.51%	94.79%	94.34%
Age at first birth	11.77%	24.11%	23.50%
Wealth index	4.52%	0.38%	− 0.96%
Age	3.75%	2.26%	2.42%
Female sterilization	3.72%	5.63%	5.79%
Age at last birth	3.19%	− 5.85%	− 5.10%
BMI	0.95%	− 1.43%	− 1.57%
Religion	0.27%	0.06%	0.05%
Health insurance	0.12%	0.45%	0.45%
Anemia	− 0.06%	− 0.12%	− 0.10%
Caste	− 0.81%	1.08%	1.05%
Area of living	− 0.85%	− 2.12%	− 2.20%
Number of children	− 4.26%	− 12.92%	− 12.86%
Contraceptive usage	− 7.14%	− 5.83%	− 6.94%
Media	0.33%		2.62%
Working status of women		− 1.83%	− 1.83%
Mobile phone usage		1.99%	2.05%
Bank accounts		0.29%	0.29%
Internet usage		− 0.95%	− 1.00%

## Discussion

The objective of this study is to analyse the changing demography of menopause in India, with specific focus on premature menopause. Our results show that 14.7% of the women in their reproductive age group (30–49 years) are in menopausal phase. This figure is higher for rural areas (15.9%) relative to urban areas (12.2%). Further, a very high proportion of women from the rural areas (about 9%) experience premature menopause in comparison to women residing in urban areas (about 5%). These figures are alarming given some previous studies on premature menopause quote that only 1 percent of women experience premature menopause^32^. Premature menopause is of greater concern as women with higher life expectancy spend a considerable amount of time in post-menopause phase^6,33,34^ needing healthcare suit their unique needs. Premature menopause is associated with increased morbidity and mortality risks for women^8,35,36^., and these women have diverse health needs^18,37–39^. Thus, our health system needs to think beyond reproductive health issues even for women in their reproductive years (15–49 years).

In terms of inter-state variations, the proportion of menopausal women varies from highest (23.9%) in Andhra Pradesh, to lowest in Manipur (10.02%). Though the proportion of menopausal women is highest in Andhra Pradesh, the proportion of women experiencing premature menopause in the age group of 30–39 years is higher in Bihar, Andhra Pradesh, Telangana, Jharkhand, Uttar Pradesh, and Karnataka. The biological factors could not solely explain these wide variations. Further, we look at the socio-demographic, nutritional and biological factors as determinants of premature menopause.

The significant predictors for premature menopause in our sample include having had hysterectomy (surgical menopause), lower levels of education, lower income quintiles, low BMI, having had a health insurance coverage, and history of female sterilization (tubectomy). Overall, our results suggest that women with lower education levels, lower nutritional status, and lower age at first and last birth are more likely to be menopausal at a premature age. Nutritional level variables, like being underweight (or low BMI) have been consistently found to be associated with premature menopause^6,20^ as nutritional deficiency is related to early ovarian degeneration and thus premature menopause. Women from lower income quintiles are expected to have lower levels of education, lower age at first and last birth as they tend to marry at a younger age and start a family early^40^. The results suggest that women from lower socio-economic backgrounds have higher odds of reaching menopause early. Similar results were reported by^6^ wherein they used NFHS-2 data, two decades back. The association of lower socio-economic background and premature menopause needs further exploration, given premature menopause has long term health hazards for women^8^.

After identifying the factors affecting the probability of premature menopause in our sample, we did decomposition analysis to understand the factors contributing to the variations in premature menopause due to varying levels of education. The decomposition analysis suggests that the majority (73%) of the inequality in premature menopause due to varying levels of education is explained by one single variable, that is “had undergone hysterectomy”. The other major factors included lower age at first and last birth, wealth index, and female sterilization. The lower age at first and last birth would predict premature menopause as Indian women consider the uterus as just a child-bearing organ and once their family is complete, they may prefer to go for uterus removal^1^. Female sterilization is highly correlated with premature menopause in our sample. Studies in the Indian context find that women who undergo tubectomy (sterilization) are more likely to undergo hysterectomy at a later age^41–43^. Few researchers have attempted to underscore the linkage between sterilization and hysterectomy. This has been linked to the quality of sterilization camps implemented in India, wherein women are prone to getting Pelvic Inflammatory Disease (PID) and need hysterectomy at a later age^42,44^. Thus, the higher incidence of sterilization among poorer Indian women could be one plausible reason for the higher prevalence of hysterectomy among them. The decomposition analysis specifically suggests that women with no education have 7% chance of premature menopause while women with higher education have only 1% chance of premature menopause. This seven-fold difference by education is primarily explained by the variable—“had undergone hysterectomy”. This indicates that women with poor education are more likely to undergo hysterectomy and that too at a younger age. This finding warrants further exploration as women with lower levels of education are more likely to be from lower socio-economic backgrounds^40^ and we would expect that women from lower socio-economic background would have limited access to surgical care, however, our results suggest otherwise. Moreover, as per the medical literature, hysterectomy should be indicated only after the conservative management options fail to give results^45,46^. Studies from India report that hysterectomy is the first line of treatment, especially for poor women with government-sponsored health insurance schemes^44,47,48^. A recent study in the Indian context^49^ examines the impact of government sponsored health insurance schemes (GSHIS) on the probability of hysterectomy among women of reproductive age group. The study finds that women with insurance coverage (GSHIS) are about 11% more likely to undergo hysterectomy and these women are from lower socio-economic background, with lower levels of education and higher order parity. The study implicitly points towards supply-side moral hazard and argues that the incentives for private hospitals empanelled under GSHIS are aligned towards prescribing more surgeries while avoiding conservative management. The issue of moral hazard (over-prescription of surgeries, or diagnostics or medicines) associated with health insurance coverage is well documented^50–53^. Studies in the Indian context found that women with lower levels of education are more likely to get trapped in supply-side moral hazard due to their lack of awareness about the side effects of surgical intervention over conservative management^54^. Studies using qualitative accounts of women belonging to poor socio-economic strata, and women having poor job security found that women have normalized hysterectomy (surgical menopause) as a solution to “excessive bleeding” due to poor or deficient sexual and reproductive health services, lack of awareness about the side-effects of hysterectomy^42,44^. The inequality in the proportion of premature menopause in our sample could also be explained by “lack of awareness about the side-effects of hysterectomy among women with lower levels of education, because of which these women could easily give into the demands of surgeons prescribing hysterectomy.

The significant association of premature menopause with socio-economic variables, like education level and income, and insurance status in our sample warrants further exploration given premature menopause increases morbidity and mortality risks for women^19,39,55,56^. Further, our results indicate that women with lower levels of education have significantly higher odds of undergoing hysterectomy relative to women with higher education. This perhaps indicates the lack of awareness and alternative treatment options for these women. Our results have implications for addressing the diverse needs of the increasing number of women in their post-menopause phase and for focusing on conservative treatment options for women with bleeding disorders at a younger age. There is a need to train medical professionals and align their incentives to promote conservative treatment and management of “excessive bleeding” over surgical intervention. Future studies are required to understand the reasons for higher rates of hysterectomy among women with lower levels of education and from lower income quintiles. The role of insurance contributing to hysterectomy at a younger age needs further exploration.

### Limitations

First, this study could not delve deeper into the reasons for premature menopause beyond surgical intervention. Perhaps the surgical intervention was required due to genuine medical reasons that are concentrated among women from low socio-economic groups. Second, the study uses the self-reported data from the NFHS-5 survey that could be susceptible to recall and social desirability bias. Perhaps future research would benefit with a mixed-method approach wherein qualitative accounts of women with premature menopause could be understood within the broader socio-cultural context of different Indian states.

## Conclusion

Using a nationally representative sample, our study finds that a very high proportion (15%) of currently married women in the reproductive age category (30–49 years) are in the menopausal phase, and this varies across Indian states. Bihar, Andhra Pradesh, and Telangana have the highest proportion of women experiencing premature menopause. The proportion of women in premature menopause varies considerably by education level. The decomposition of the predictors of premature menopause by education level suggests that hysterectomy is the major contributor to the inequality in the proportion of premature menopause by education level. There is a need to further explore reasons for women opting for hysterectomy at a younger age given premature menopause at a younger age have implications for morbidity and mortality risks for women in their later years.

## Data Availability

The study utilizes secondary sources of data that are freely available in the public domain through https://dhsprogram.com/methodology/survey/survey-display-541.cfm. Those who wish to access the data may register at the above link and thereafter can download the required data free of cost.
